# The Hepatic Stellate Cells (HSTCs) and Adipose-derived Mesenchymal Stem Cells (ASCs) Axis as a Potential Major Driver of Metabolic Syndrome – Novel Concept and Therapeutic Implications

**DOI:** 10.1007/s12015-021-10304-w

**Published:** 2021-11-25

**Authors:** Krzysztof Marycz, Katarzyna Kornicka-Garbowska, Larry Galuppo, Lynda Bourebaba

**Affiliations:** 1 Department of Experimental Biology, Faculty of Biology and Animal Science, Wrocław University of Environmental and Life Sciences, Norwida 27B, 50-375 Wrocław, Poland; 2 International Institute of Translational Medicine, Jesionowa 11, Malin 55-114, Wisznia Mała, Poland; 3 Department of Surgical and Radiological Sciences, School of Veterinary Medicine, University of California Davis, Davis, CA USA

**Keywords:** HSTCs, ASCs, A-Reg, Hepatokines, Adipokines, Metabolic syndrome, Crosstalk

## Abstract

**Abstract:**

Herein, we would like to introduce a novel concept for the prevention and treatment of metabolic syndrome, which is based on molecular relationship between liver and adipose tissue. Particularly, we believe, that unravelling the molecular crosstalk between hepatokines and adipokines will allow to better understand the pathophysiology of metabolic diseases and allow to develop novel, effective therapeutic solutions against obesity and metabolic syndrome.

**Graphical Abstract:**

Inter-organ communication on the level of stem progenitor cells-hepatic stellate cells (HSTCs) and adipose-derived progenitors (ASCs) could represents a key mechanism involved in controlling glucose tolerance as well as insulin sensitivity.

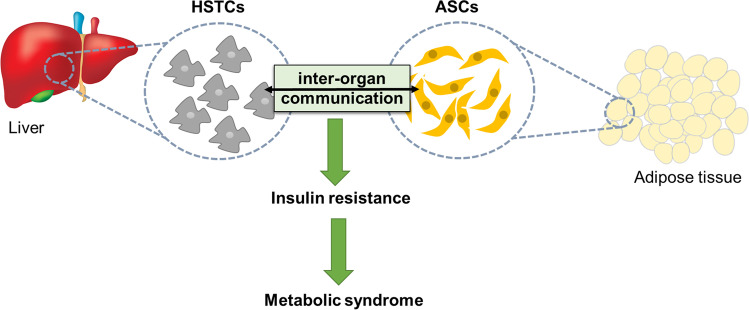

## Introduction

Metabolic syndrome (MetS) in humans is defined as an clusters of metabolic abnormalities including insulin resistance (IR), hyperglycemia, dyslipidemia, obesity or atypical regional adiposity[1]. A similar disease in horses-equine metabolic syndrome’ (EMS), resembles what we know from MetS in abovementioned aspects. Distinct differences relate to vascular structures, MetS affects mainly coronary vessels while EMS are prone to laminitis. To underline that clinical issue, it is worth to remember that one of the best racing horses in our history, including Secretariat and 2006 Kentucky Derby winner Barbaro, were euthanized due to laminitis. Thus, we have irretrievably lost our unique legacy due to metabolic related disorders. Similarly, humans’ cardiovascular complications are recognized as the leading cause of death in well-developed countries all over the world. Nowadays, while sedentary life style, high calory food and lack of exercise become more common, the threat for metabolic disorders development rapidly increased. Such a lifestyle is globally recognized as a risk factor for obesity, insulin resistance, MetS or EMS. It was shown that cardiovascular events are diagnosed 7 times more often in MetS patients compared to non- MetS individuals [2]. Such data does not exist for horses; however, it might be speculated with high degree of probability, that EMS becomes critical and fundamental risk factor of laminitis. Therefore, due to its burden, development of effective therapeutic strategies against MetS and EMS is urgently required. In general, pharmaceutical or nutraceutical therapies in the course of MetS are based on body weight reduction and glucose homeostasis restoration strategies. However fundamental question is to ask where are the gaits opening these possibilities. Some focus on the development of inhibitors and activators that target adipose tissue insulin receptor or insulin receptor substrate. Other approach utilize mesenchymal stem cells injections which showed great improvement in body weight, glucose tolerance and dyslipidemia [3, 4]. In the light of recent research, skeletal muscle-liver-fat axis controls organismal energy distribution and thus represent a potential target for the development of therapies against various metabolic disorders [5]. Many recent excellent research papers proposed the concepts for targeting particular receptors on liver cells, adipocytes or other cells in order to improve insulin sensitivity.

However, we state, that the rules and factors that drive inter-organ communication between liver and adipose tissue should be discovered and described in order to fully understand pathophysiology of metabolic disorders. Herein, we would like to introduce a concept that assumes the understanding of inter-tissue communication, in order to alleviate metabolic diseases incidence and develop effective therapeutics strategies.

## Why Did we Decide to Present That Concept?

From several years our group alone and in cooperation with others researchers from all over the world are searching for an effective therapeutic solution for treatment of EMS and MetS. We focus on the molecular and physiological role of stem progenitor cells in the course of EMS and MetS development and their unquestionable role in insulin resistance initiation. Our previous studies showed, that systemic administration of autologous stem cells improves insulin sensitivity, however the clinical effect expires over a short period of time after their administration [6]. For that reason, we state, that this short term positive clinical effect could be prolonged but in “this game, an additional player is required”. Therefore, we would like to propose a novel concept in which liver-adipose tissue progenitor cells are a critical stem cells population that might become a therapeutic target for a new treatment of EMS or MetS. Searching for particular hepatokines, that could affect both hepatic stellate cells (HSTCs) and adipose-derived mesenchymal stem cells (ASCs) might become a groundbreaking therapeutic solution for EMS and MetS treatment.

## Hepatokines Affect Adipose-derived Mesenchymal Stem Cells (ASCs) Properties

Systemic metabolism in humans and other higher species is governed by a number of complex pathways, that mainly regulate energy expenditure and nutrients intake. Liver is considered as being a major metabolism regulatory organ, that maintains energy homeostasis through the detection of nutrients afflux variations and the adjustment of energy and metabolites production balance required by other tissues [7]. The importance attributed to the liver as being at the center of metabolic regulation also stems from its ability to communicate with other key tissues including CNS, adipose tissue, and skeletal muscle. This crosstalk is mainly driven by the production of a number of important signaling molecules known as hepatokines, which have been reported to be essential for transmitting information regarding the metabolic status of the liver. To this end, various hepatokines have been demonstrated in recent years to be closely involved in obesity, insulin resistance, and NAFLD development, through their effects on neighboring tissues, notably adipose tissue and its resident cells [8]. While sustained altered expression levels of certain hepatokines has been proposed as a reliable metabolic dysfunction indicator, expression of other hepatokines has been shown to dynamically fluctuate with physiological states changes, denoting their critical involvement in metabolic balance uphold [9]. By way of example, fetuin A, adropin as well as fibroblast growth factor 21 (FGF21) hepatokines have been demonstrated to be involved either positively or negatively in insulin sensitivity, inflammation and thermogenesis regulation of target organs, including adipose, bone and heart [10]. Fibroblast growth factor-21 (FGF21) represents one of the most common liver-derived hormones, that positively regulate human metabolism and energy homeostasis. FGF21 predominantly acts on glucose and lipids metabolism by increasing the oxidation of fatty acids as well as insulin sensitivity, while decreasing gluconeogenesis in liver [11]. In adipose tissue, liver FGF21 has been demonstrated to substantially modulate lipolysis in both *in vivo* and *in vitro* models, whereas stimulating glucose uptake in human adipocytes through the GLUT1 and adiponectin expression increment. Moreover, elevated FGF21 levels that correlates with obesity, have been attributed to the establishment of an adaptive mechanism for the mitigation of insulin resistance and its metabolic consequences [12]. FGF21 has also been implicated in the regulation of cell longevity thus, previous study underscored that FGF21 hepatokine modulates MSCs senescence via the regulation of mitochondrial dynamics. Thereby, the investigation’s outcomes showed that in the presence of FGF21, the MSCs displayed decreased expression of p21 and p53 proapoptotic factors, together with reduced ROS generation, enhanced Mfn2 induced mitochondrial fusion, as well as decreased p-Drp1 protein, all of which have been attributed to inducement of the AMPK signaling by FGF21 hepatokine [13]. Since FGF21 is also well recognized for its anti-inflammatory properties in obesity related inflammation, *Kang and colleagues*, demonstrated that combination of FGF21 with adipose-derived mesenchymal stem cells (ASCs) considerably improved their anti-inflammatory and antifibrotic properties in an *in vitro* mouse model of thioacetamide-induced liver fibrosis. Thus, the presented results clearly indicated that FGF21_ASCs significantly inhibited TGF‐β‐induced expression of fibrogenic factors, including hyaluronic acid, α‐smooth muscle actin (α‐SMA), collagen and tissue inhibitor of metalloproteinase‐1 (TIMP‐1), mainly over the repression of p‐JNK, NF‐κB and p‐Smad2/3 signaling. This research additionally pointed out that FGF21 stimulated ASCs produced higher amounts of α‐lactoalbumin (α‐LA) and lactotransferrin (LTF) secretory factors, known for their liver protective and anti‐fibrotic effects [14].

Adropin is another important small peptide primarily secreted by the liver. The circulating hepatokine levels are usually subjected to variations according to the energy status and nutritional content of the diet. Moreover, low levels of Adropin have been reported in patients affected by either obesity, fatty liver, insulin resistance, and CVD, suggesting the existence of a crosstalk between adropin functions and metabolic disorders [15]. Adropin has been reported to regulate body weight as well as lipid and glucose homeostasis. As a matter of fact, previous investigation demonstrated that adropin hepatokine is also implicated in adipogenesis control in white fat. Accordingly, a strong stimulation of fat precursor cell proliferation via a ERK1/2-dependent mechanism following liver adropin treatment has been observed; furthermore the recorded suppressed adipogenic and lipogenic genes expression including *Pparγ*, *Fabp4*, *C/ebpα* and *Fasn*, as well as intracellular lipid accumulation during rat and mouse preadipocytes differentiation, strongly suggests that this hepatokine may participate in white adipogenesis control in dysregulated adipose tissue [16]. Impaired subcutaneous adipogenesis, including abnormal ASCs differentiation and expansion, has been associated with obesity and obesity-associated metabolic abnormalities such as dysfunctional, hypertrophic adipocytes, chronic low-grade inflammation, and peripheric insulin resistance [17]. Previous research has defined that obese ASCs were prone to uncontrolled and inappropriate adipogenesis, thereby worsening the adipose tissue expansion volume during obesity onset; this tendency has been mainly attributed to the characteristic inflammatory microenvironment of obese adipose tissue, and to the resulting overexpression of TNF-α [18]. Taken together, it is reasonable to postulate that similarly to preadipocytes, liver-derived adropin may positively impact adipose tissue residing progenitor cells, and thus attenuate aberrant adipogenic differentiation and related adipocyte hyperplasia and hypertrophy. It is also probable that observed collapsed adropin levels in obese subjects may contribute partly to the uncontrolled adipogenic maldifferentiation of ASCs cells in the course of obesity. Leukocyte cell-derived chemotaxin 2 (LECT2), is another example of hepatokine which is critically involved in metabolic disruption. This neutrophil chemotactic protein, which is essentially produced by the liver, has already been linked with the development of sepsis, liver disease, metabolic syndrome, arthritis and cancer [19]. Indeed, increased liver production of LECT2 have been related to obesity development in rats, and resulting metabolic stress, which subsequently impairs insulin signaling, promote adhesion molecules expression, and increases pro-inflammatory cytokines synthesis [15]. Lately, *Jung and collaborators* evaluated in their investigation, the impact of LECT2 on inflammation and insulin resistance in differentiated 3T3-L1 adipocytes. They observed that LECT2 induced an NF-κB-mediated pro-inflammatory response, characterized by significant stimulation of pro-inflammatory cytokines namely, TNFα and MCP-1. Likewise, treated cells displayed upregulated lipogenic SREBP1c and SCD1 genes, accompanied by lipid overaccumulation in pre-adipocytes, and simultaneous breakdown of insulin receptor substrate (IRS-1) and Akt phosphorylation, indicating that LECT2 hepatokine greatly contributes to adipose tissue obesity, insulin resistance and inflammation through CD209/P38-dependent signaling and activation of downstream molecules [20]. According to several reports, ASCs derived from patients with morbid obesity exhibit amplified secretion levels of various pro-inflammatory cytokines like IL-6, IL-1β, IL-17 and TNF-α, diminished immunomodulatory ability, impaired proliferation, clonogenicity and suppressed expression of stemness and differentiation related genes, which denote the critical potential of obesity to elicit molecular alterations in ASCs to promote chronic low-grade systemic inflammation, and ultimately failure in adipocyte insulin response [21–23]. On the basis of all these interrelated findings, it is quite conceivable that LECT2 hepatokine in the framework of liver-adipose tissue crosstalk, may accentuate the ASCs inflammatory character, and therefore trigger to the chronic low-grade inflammation exacerbation of obese adipose tissue, and thereby at long last lead to the onset of insulin resistance and hence generalized metabolic failure of adipose tissue.

## The Hepatic Stellate Cell (HSTCs) and Adipose Derived Mesenchymal Stem Cells (ASCs) Axis

Adipose tissue (AT) and liver play crucial role in maintaining organism energy homeostasis, but they lack protective mechanisms against nutrient overload. Due to chronic metabolic stress during obesity, these endocrine organs become triggering factors for diabetes, MetS and EMS development. For many years, AT and liver implications in development of endocrine disorders was studied separately. However, in the light of recent evidences, holistic and complex approach should be applied in order to unravel the crosstalk between AT and liver actions.

As a consequence of nutrient overload and development of obesity, adipocytes enlarge and fail to store excessive amount of lipids, which are redirected to other organs, mainly liver, in which they trigger lipotoxicicty and subsequently insulin resistance. Recent evidence has suggested, that chronic inflammation and hypertrophy negatively impacts adipose progenitor stem cells within AT, which worsens IR and general metabolic status of the organism. This observation initially came from the fact, that ASCs release grate number of bioactive factors and directly regulate multiple functions in AT. However, recent data clearly indicated on the heterogeneity of ASC pool suggesting that their distinct phenotypes exert different functions *in vivo*. Therefore, ASC should not be treated as uniform stem cells pool and prior future studies should be categorized into selected subpopulations in accordance to their unique complements of surface antigens. Study performed by Raajendiran et al. [24] identified three molecularly distinct subpopulation of ASC characterized by CD34+ high (CD34^high^), low (CD34^low^) and no (CD34^−^) expression. Strikingly, CD34^high^ derived adipocytes were characterized by high rate of lipolysis and fatty acid storage while CD34- gave rise to beige-like adipocytes. What is more, T2D patients were characterized by increased in CD34^high^ and decreased CD34- cells, which indicates that deterioration in ASCs composition correlates with development of metabolic disease. These noteworthy data was extended by the findings of Schwalie and colleagues [25] who discovered CD142+ ASC subpopulation termed as adipogenesis-regulatory cells (A-Regs), which suppress adipocyte differentiation via paracrine mechanism. As these cells modulate AT plasticity, their deterioration might be directly involved in the development of metabolic disorders by unlashing the abundant proliferation of CD34^high^. Affecting A-Regs functionality can come from intrinsic microenvironment or even different tissue/organs including liver. For that reason, there is an urgent need to identify the biological agents and molecular pathways modulating A-Regs functions. Unravelling the crosstalk between distinct cells populations and their secretom, was shown to be crucial for understanding of pathological mechanisms driving endocrine disease development. Understanding A-Regs cytophysiological properties may open new possibilities for new therapeutic strategies against T2D, MetS and EMS.

Disproportion of ASCs subtypes equals to disproportion in biological agents, which they and their progeny secrete. Multiple adipokines and cytokines have been implicated in the development of obesity, MetS and EMS and several of them directly affects not only AT but also liver. Among them, leptin was shown to control crucial aspects of liver metabolism e.g. insulin resistance, lipotoxicicty and fibrosis by the activation of quiescent hepatic stellate cells (HSTCs). Both, autocrine and paracrine stimulation activates proliferation of HSTCs, which becomes resistant to apoptosis [26] and promote liver fibrosis. Alternation of messengers by which AT and liver communicate leads to disruption of body homeostasis and predispose to development of metabolic disease. Modulation of distinct cell population in these two organs may help restore that balance. For example, targeting A-Regs may inhibit abnormal CD34^high^ proliferation, decrease leptin level and HSTCs dis-activation protecting the liver against metabolic influx. On the other hand, identification of hepatokines that target and deteriorate the metabolism of different ASCs subtypes may help to restore AT homeostasis and prevent disease progression. Finding key messengers in that inter-organ communication is absolutely necessary to gain complete understanding of pathophysiological process and thus develop effective therapeutic strategies.

We believe that adipose tissue, particularly ASCs, are a critical driver of liver impairment during metabolic disorders. Both, AT and liver secrete biological factors, which modulate both of these organ’s metabolism. Identification of the key players in that inter-organ communication will allow to understand why AT-liver axis represents a gatekeeper for metabolic health. Multidirectional interactions between tissues maintains body homeostasis but alternation in hormones and cytokines, key messengers in that specific information exchange, disrupts the network and contributes to the development of metabolic disorders. Understanding of AT-liver crosstalk is critical to redefine therapeutic strategies targeting metabolic syndrome.

## Summary

The global pandemic of metabolic syndrome requires the urgent development of effective therapeutic strategies. For that reason, here we presented our novel concept in which inter-organ communication on the level of stem progenitor cells (hepatic stellate cells – adipose-derived progenitors) could represents a key mechanism involved in controlling glucose tolerance as well as insulin sensitivity. Both adipose tissue and liver are critically involved in glucose tolerance and obesity development, which are recognized as an initiating factor for metabolic disorders development. More specifically, we hypothesized, that the existing paracrine crosstalk between hepatic stellate cells and A-regs may finally reinforce insulin sensitivity and improve glucose tolerance. We further reason that finding a paracrine mechanism that regulates metabolic activity of liver stellate cells and adipogenesis regulatory cells will open a new avenue for developing effective therapeutic solution for metabolic syndrome.

## Data Availability

Not applicable.
